# Antibiotic treatment for appendicitis in Norway and Sweden: a nationwide survey on treatment practices

**DOI:** 10.1186/s12893-022-01680-2

**Published:** 2022-06-15

**Authors:** M. V. Gran, D. Kjønås, U. Gunnarsson, K. Strigård, A. Revhaug, E. K. Aahlin

**Affiliations:** 1 Department of Gastrointestinal Surgery, St. Olavs Hospital, Trondheim University Hospital, Trondheim, Norway; 2 Department of Gastrointestinal and HPB Surgery, University Hospital of Northern Norway, Tromsø, Norway; 3 Department of Surgical and Perioperative Sciences, Surgery, Umeå University, Umeå, Sweden; 4 Department of Clinical Medicine, Faculty of Health Sciences, UiT The Arctic University of Norway, N-9037 Tromsø, Norway

**Keywords:** Appendicitis, Conservative treatment, Surgery, Surveys and questionnaires, Anti-bacterial agents, Appendectomy

## Abstract

**Background:**

Appendicitis is one of the most common causes of acute abdomen. Uncomplicated appendicitis is as an inflamed appendix without perforation, gangrene or abscess formation. Recent trials show that one can safely treat uncomplicated appendicitis with antibiotics, given patient approval and appropriate follow-up. A recent study has also indicated no difference between antibiotic treatment and placebo. Our aim was to investigate if Norwegian and Swedish surgical departments treat uncomplicated appendicitis with antibiotics and to explore their opinions on this treatment practice.

**Methods:**

A questionnaire was distributed to all heads of department in hospitals that treat appendicitis in Norway and Sweden. Answers were collected using a REDCap survey. Answers were compared between centers and nations and the results were presented anonymously.

**Results:**

We sent the questionnaire to 94 eligible recipients and received 61 (65%) answers. In total, 8/61 (13%) departments stated that they have established antibiotic treatment as sole treatment for uncomplicated appendicitis. Almost half of the responders stated that they have used antibiotics sporadically to treat uncomplicated appendicitis. Lack of evidence and guidelines were noted as reasons why antibiotic treatment has not been implemented as sole treatment.

**Conclusions:**

Most Norwegian and Swedish departments have not implemented antibiotic treatment as the sole treatment for uncomplicated appendicitis. Despite several recent large trials on this subject, lack of evidence and guidelines was the most frequently reported reason in our survey.

## Background

Appendicitis is one of the most common causes of acute abdomen. The incidence rate in Northern Europe is estimated to 1 per 1000 person years [1]. The lifetime incidence of appendicitis is reported to be between six and nine percent. While the incidence has decreased over the past decades, appendicitis still remains the most common identifiable cause of hospital admissions due to abdominal pain in the western world [2, 3].

Complicated appendicitis is defined as intraoperatively or histologically confirmed perforated, suppurative or gangrenous appendicitis or appendicitis with an abscess or peri-appendicular mass. Uncomplicated appendicitis refers to acute appendicitis without these factors and represents approximately 70% of all cases of acute appendicitis [3].

The first successful appendectomy was performed on December 6, 1735 in London by the French surgeon Cluadius Amyand on an 11-year old boy whose appendix was punctured by a pin that he had swallowed [4]. Now, appendectomy is the established treatment for appendicitis and has become the most common emergent surgical procedure [5]. Considering how frequent patients present due to suspected appendicitis, diagnostic pathways for this illness is still a major topic for discussion. Clinical features such as fever with migratory abdominal pain are often associated with acute appendicitis. Patients will frequently present with elevated leucocyte counts combined with elevated C-reactive protein levels. Authors debate whether clinical scoring systems are sufficient for diagnosing acute appendicitis. Even if they may not establish an accurate diagnosis, these scoring systems have proved to be useful in reducing the use of imaging, hospital admissions and negative surgical explorations. For instance, an Alvarado-score of less than 5 is considered sufficient to exclude acute appendicitis without the need for additional imaging [7]. Some authors also propose that high Alvarado-scores have the same positive likelihood ratio for diagnosing acute appendicitis as that of computed tomography (CT). Other authors have also claimed that focused ultrasonography delivers high positive- and negative predictive values. The Jerusalem guidelines therefore recommend using clinical parameters and ultrasound to reduce the need for CT [8]. In separating uncomplicated versus complicated appendicitis, low dose CT has been proven non-inferior to standard dose-imaging [8].

In the last decades there have been several randomized trials and meta-analyses investigating if uncomplicated appendicitis can be treated conservatively with broad-spectrum antibiotics [9–15]. There is also evidence suggesting that uncomplicated appendicitis might be a spontaneously resolving illness, a claim supported by Park and colleagues’ randomized controlled trial published in 2017 [16]. This also correlates to observations made during McBurney’s time, when surgeons suggested that not all cases of appendicitis needed surgery and obviously no antibiotics were available at that time [17]. The aim of this study was to investigate if surgical departments in Norwegian and Swedish hospitals treat uncomplicated appendicitis conservatively with antibiotics and to establish possible differences between the two countries, as well as to investigate their general opinion on this issue. Has new evidence led to a change in clinical practice?

## Methods

The authors designed and distributed a questionnaire to all heads of department in hospitals treating appendicitis in Norway and Sweden. The questionnaire was distributed during the fall of 2018. Departments that treat acute surgical conditions were determined as sites where appendicitis is treated. We identified 94 surgical departments with an acute commission, 41 in Norway and 53 in Sweden. Heads of department were identified by either knowing their name, calling their department or by information provided on their hospital website. In cases where adequate contact details were missing, we sent the survey to the hospital’s general email, addressed to the head of the surgical department. Two of the recipients replied two times each, with different answers each time. In these cases, only their initial response was registered.

The questionnaire was sent out using a REDCap (Vanderbilt, 2004) survey linked to the email. It included questions with the possibility to select multiple alternatives, as shown in Table 1.

**Table 1:** Copy of the multiple-choice questionnaire

1. Has your department established antibiotic treatment as a single treatment modality for uncomplicated appendicitis?
a. Yes, oral treatment
b. Yes, intravenous treatment
c. No
2. Has your department treated patients with uncomplicated appendicitis sporadically/unsystematically with antibiotics?
a. Yes, oral treatment
b. Yes, intravenous treatment
c. No
3. Why has your department not implemented antibiotic treatment for uncomplicated appendicitis?
a. Not relevant, we routinely use antibiotics as a single treatment modality
b. The available documentation is too weak
c. Lack of guidelines
d. Concern regarding developing resistance to antibiotics
e. Patients prefer surgical treatment
f. Surgeons prefer surgical treatment Antibiotic treatment as a single treatment modality relates to increased use of radiological investigations (computed tomography and ultrasound)
g. Other causes
4. Would your department participate in a multicenter study regarding antibiotic treatment for uncomplicated appendicitis?
a. Yes
b. No
c. Do not know
d. We already participate in an ongoing study

More than one answer could be registered

The results were analyzed using absolute numbers with percentages. Statistical significance was tested using a two-sample Students t-test. P-values < 0.05 were considered significant. Statistical analysis was performed and figures were created using a spreadsheet (Microsoft Corporation, 2010. Microsoft Excel).

The authors received no funding in relation to this survey. There are no competing interests.

All methods were in accordance to relevant guidelines and regulations. This survey did not obtain sensitive information, so that ethical approval or consent was not required.

## Results

We received 61 replies out of 94 eligible recipients (65% response rate). In Norway, 32 out of 41 recipients replied (78% response rate) while 29 out of 53 replied in Sweden (55% response rate).

**Fig. 1:**
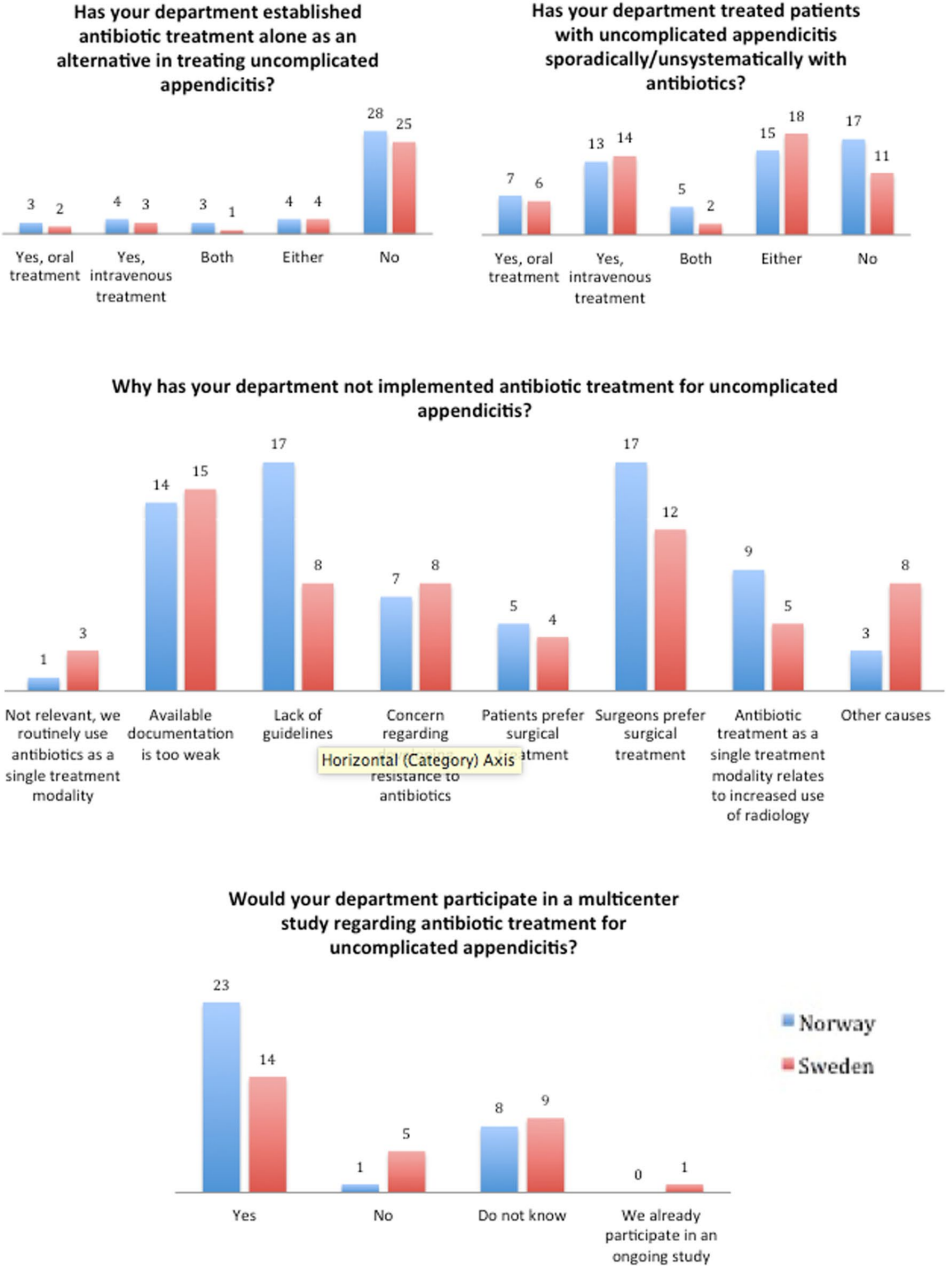
Bar graph, using absolute numbers, illustrating the response from the questionnaire. Norwegian response in blue. Swedish response in red

In total, 8 out of 61 (13%) departments replied that they had established either oral or intravenous antibiotic treatment as a solitary treatment for acute appendicitis. Approximately half of the replying departments stated that they have treated acute appendicitis sporadically/unsystematically with antibiotics. Almost half of the departments (48%) answered that the available documentation on antibiotic treatment is too weak. Nine (15%) departments replied that patients prefer surgical treatment, while 29 (48%) felt that surgeons prefer surgical treatment. More than half of the departments (61%) replied that they would like to participate in a future study aimed to determine the clinical value of antibiotic treatment for acute appendicitis. Six departments do not wish to participate; one department is currently participating in an ongoing study, while 17 replied that they do not know. Table 2 and Fig. 1 illustrate the answers and differences between Norwegian and Swedish replies. As shown in Table 3, when not distinguishing between oral or intravenous and sporadic or systematic treatment, a higher percentage of Swedish (62%) than Norwegian (47%) departments have used antibiotics for treating uncomplicated appendicitis, but there was no statistically significant difference.

**Table 2:** Response from the questionnaire shown in Table 1

*Has your department established antibiotic treatment alone as an alternative in treating uncomplicated appendicitis?*			
	Norway	Sweden	Total^1^
Yes, oral treatment	3 (9%)	2 (7%)	5 (8%)
Yes, intravenous treatment	4 (13%)	3 (10%)	7 (11%)
Both	3 (9%)	1 (3%)	4 (7%)
Either	4 (13%)	4 (14%)	8 (13%)
No	28 (87%)	25 (86%)	53 (87%)
*P-value*	*0.84*		

*Has your department treated patients with uncomplicated appendicitis sporadically/unsystematically with antibiotics?*			
	Norway	Sweden	Total
Yes, oral treatment	7 (22%)	6 (21%)	13 (21%)
Yes, intravenous treatment	13 (40%)	14 (48%)	27 (44%)
Both	5 (16%)	2 (7%)	7 (11%)
Either	15 (47%)	18 (62%)	33 (54%)
No	17 (53%)	11 (38%)	28 (46%)
*P-value*	*0.75*		

*Why has your department not implemented antibiotic treatment for uncomplicated appendicitis?*			
	Norway	Sweden	Total
Not relevant, we routinely use antibiotics as a single treatment modality	1 (3%)	3 (10%)	4 (7%)
Available documentation is too weak	14 (44%)	15 (52%)	29 (48%)
Lack of guidelines	17 (53%)	8 (28%)	25 (41%)
Concern regarding developing resistance to antibiotics	7 (22%)	8 (28%)	15 (25%)
Patients prefer surgical treatment	5 (16%)	4 (14%)	9 (15%)
Surgeons prefer surgical treatment	17 (53%)	12 (41%)	29 (48%)
Antibiotic treatment as a single treatment modality relates to increased use of radiological investigations (CT/US)	9 (28%)	5 (17%)	14 (23%)
Other causes	3 (9%)	8 (28%)	11 (18%)
*P-value*	*0.64*		

*Would your department participate in a multicenter study regarding antibiotic treatment for uncomplicated appendicitis?*			
	Norway	Sweden	Total
Yes	23 (72%)	14 (48%)	37 (61%)
No	1 (3%)	5 (17%)	6 (10%)
Do not know	8 (25%)	9 (31%)	17 (28%)
We already participate in an ongoing study	0	1 (3%)	1 (2%)
P-value	0.90		

Regarding question 1 and 2: Responders who selected alternatives a + b are also shown as “both” and responders who selected either a or b are also shown as “either”. Answers in Norway and Sweden combined are shown as “total”. P-value calculated using a Students t-test

^1^Norway and Sweden combined

P-value for the differences between Norway and Sweden

**Table 3:** Summary of response from question 1 and 2

*Surgical departments which have used oral or intravenous antibiotics, systematically or sporadically, when treating uncomplicated appendicitis*			
	Norway	Sweden	Total
Yes	15 (47%)	18 (62%)	33 (54%)
No	17 (53%)	11 (38%)	28 (46%)
P-value			0.80

P-value calculated using a Students t-test

P-value for the differences between Norway and Sweden

When asked why antibiotic treatment was not implemented, the risk of recurrent appendicitis, the risk of missing an appendiceal tumor, and the need for CT was noted. One department also replied that they do not treat uncomplicated appendicitis with antibiotics in cases where surgery is not performed.

## Discussion

The main purpose of this study was to investigate conservative treatment with antibiotics for acute uncomplicated appendicitis in Norway and Sweden. We found that more than half of the responding surgical departments have used antibiotics for uncomplicated appendicitis. Some departments have implemented antibiotics as routine treatment. Conservative treatment seemed to be used more frequently in Sweden than Norway, but more sporadically than as a routine practice. Several responders noted the lack of scientific evidence, lack of guidelines and surgeon preference as reasons why antibiotic treatment was not implemented. To our knowledge, our survey is the first nationwide study on treatment of appendicitis in both Norway and Sweden, where guidelines for antibiotic treatment, as the sole treatment for uncomplicated appendicitis, are currently not available. The distributed survey included four questions which were intended to give an overview of the treatment practices in these two countries. This might be a weakness of this study, as more detailed information regarding the use of antibiotics will not be present. We felt, however, that a short and concise questionnaire would deliver a higher response rate.

Non-operative management of uncomplicated appendicitis has been evaluated in several randomized trials. The APPAC trial, a multicenter randomized-controlled trial compared antibiotic therapy with appendectomy in the treatment of uncomplicated, CT-proven acute appendicitis in 530 patients [10]. After 1-year follow-up, 70 out of 256 patients (27%) in the antibiotic group had undergone appendectomy due to either treatment failure or recurrence. These findings did not meet the non-inferiority level set in this study. Long-term results from the APPAC trial showed that among patients treated with antibiotics, 39% suffered recurrent appendicitis within 5 years. Of those patients who underwent surgery for recurrence, 2% had complicated appendicitis. The overall complication rate was deemed favorable in the antibiotic group [18]. Similarly, in an RCT of 243 patients, Vons et al. found that antibiotic management was not non-inferior to surgical treatment [8]. However, like the APPAC trial, several previous studies have demonstrated that antibiotic treatment is a good alternative to surgery in patients with uncomplicated acute appendicitis [9, 12, 14, 15]. At present, there is no consensus on whether conservative treatment is considered equal to appendectomy in cases of uncomplicated appendicitis. However, based on the findings from previous studies, treatment algorithms including conservative management has been suggested. The main issue might be to determine which patients will benefit from conservative management, and if conservative management entails antibiotics or simply supportive treatment. In response to these questions, there are ongoing studies in Finland aimed to further our knowledge on this disease. The APPAC II trial aims to determine if oral antibiotics are as safe to use as a regime of intravenous followed by oral antibiotics, as used in the first APPAC trial [19]. In addition, the APPAC III trial will evaluate antibiotics against placebo in a double-blind superiority randomized trial [20].

Our results show that when analyzing both countries together, 8 out of 61 (13%) had antibiotics as an established treatment modality. This number might seem low compared to the number of departments that have used antibiotics sporadically, but considering the lack of international consensus, established guidelines and the actual proof of antibiotics being superior to only supportive treatment, one might suggest that this low number could be explainable. Using antibiotics sporadically/unsystematically seems to have been more prevalent in Sweden. There are relatively few university hospitals in both Norway and Sweden, so one could not establish any significant differences between University and non-University affiliated hospitals. More Norwegian responders were concerned about the lack of guidelines as to why they have not implemented antibiotic treatment. It is therefore interesting to note that more Norwegian departments are willing to participate in future studies than in Sweden (72% vs 48%). This might suggest that this topic has been more frequently discussed and tested in Sweden, as shown by a higher rate of sporadic treatment with antibiotics in that country. It is therefore interesting to note that despite numerous studies and discussions on this topic, we have yet to establish a multi-center treatment guideline or consensus regarding treatment of uncomplicated appendicitis, as shown by the results in question 1 and 2.

During most of the twentieth century, the belief was that all cases of appendicitis would eventually result in perforation if appendectomy was not performed within a short time. Appendectomy therefore became more or less a prophylactic intervention to reduce mortality [17, 21]. This disputes the basis of McBurney’s 1889 report, in which he sought to find a safe surgical treatment for those patients presenting with deteriorating illness [22, 23]. In addition, a recent meta-analysis has shown that one can safely observe patients with diagnosed uncomplicated appendicitis for up to 24 h before surgery [24]. It is therefore reasonable to suspect that uncomplicated appendicitis does not necessarily demand appendectomy, as perforation is not a predestined outcome.

Surgical treatment is, however, still the recommended treatment for uncomplicated appendicitis. Some studies have suggested antibiotic treatment as being a safe option, but the role of antibiotics is also disputed as recent evidence show that uncomplicated appendicitis might be a self-limiting disease. Finally, one must also note concerns regarding increasing bacterial resistance to antibiotics. One might argue that this issue should be addressed in future studies, as the effect (or even necessity) of broad-spectrum antibiotics for uncomplicated appendicitis is not well established.

## Conclusions

Some surgical departments have established antibiotic treatment as an alternative in treating uncomplicated appendicitis, but many departments seem to find the lack of evidence concerning. There are few differences in how Norwegian and Swedish surgical departments view the treatment of uncomplicated appendicitis. Several Norwegian and Swedish surgical departments have stated that they would participate in a study on conservative treatment of appendicitis.

## Data Availability

All data generated or analyzed during this study are included in this published article.

## Abbreviations

CT: Computed tomography
RCT: Randomized controlled trial
